# Diving Response in Rats: Role of the Subthalamic Vasodilator Area

**DOI:** 10.3389/fneur.2016.00157

**Published:** 2016-09-21

**Authors:** Eugene V. Golanov, James M. Shiflett, Gavin W. Britz

**Affiliations:** ^1^Department of Neurosurgery, The Houston Methodist Hospital, Houston, TX, USA; ^2^Department of Neurosurgery, University of Mississippi Medical Center, Jackson, MS, USA

**Keywords:** diving response, nasal ammonia stimulation, forehead cold stimulation, autonomic parameters, neuroprotection, subthalamic vasodilator area, electroencephalography

## Abstract

Diving response (DR) is a powerful integrative response targeted toward survival of the hypoxic/anoxic conditions. Being present in all animals and humans, it allows to survive adverse conditions like diving. Earlier, we discovered that forehead stimulation affords neuroprotective effect, decreasing infarction volume triggered by permanent occlusion of the middle cerebral artery in rats. We hypothesized that cold stimulation of the forehead induces DR in rats, which, in turn, exerts neuroprotection. We compared autonomic [AP, heart rate (HR), cerebral blood flow (CBF)] and EEG responses to the known DR-triggering stimulus, ammonia stimulation of the nasal mucosa, cold stimulation of the forehead, and cold stimulation of the glabrous skin of the tail base in anesthetized rats. Responses in AP, HR, CBF, and EEG to cold stimulation of the forehead and ammonia vapors instillation into the nasal cavity were comparable and differed significantly from responses to the cold stimulation of the tail base. Excitotoxic lesion of the subthalamic vasodilator area (SVA), which is known to participate in CBF regulation and to afford neuroprotection upon excitation, failed to affect autonomic components of the DR evoked by forehead cold stimulation or nasal mucosa ammonia stimulation. We conclude that cold stimulation of the forehead triggers physiological response comparable to the response evoked by ammonia vapor instillation into nasal cavity, which is considered as stimulus triggering protective DR. These observations may explain the neuroprotective effect of the forehead stimulation. Data demonstrate that SVA does not directly participate in the autonomic adjustments accompanying DR; however, it is involved in diving-evoked modulation of EEG. We suggest that forehead stimulation can be employed as a stimulus capable of triggering oxygen-conserving DR and can be used for neuroprotective therapy.

## Introduction

“Diving response” (DR) is a specialized integrative state of the organism targeted toward survival of potentially hypoxic/anoxic conditions, such as diving (1). Observed in diving animals, archetypal DR consists of the coordinated activation of at least three reflexes: simultaneous activation of parasympathetic and sympathetic systems and respiratory adjustments (2, 3). Activation of these reflexes leads, respectively, to bradycardia (4–10), peripheral vasoconstriction limiting blood supply to muscles and “non-critical” organs (2, 11–15), increase in arterial pressure (2, 16–18), and apnea (12, 18, 19).

Diving response is initiated by the excitation of the ophthalmic division of the trigeminal nerve (2), which innervates nasal mucosa, cornea, forehead, and cerebral dura mater (20, 21). Stimulation of the nasal mucosa by perfusion of water or ammonia vapors through the nasal cavity triggers similar response (6, 22). Direct stimulation of the ethmoidal nerve branching from the ophthalmic division and innervating the nasal mucosa produces DR (23).

Autonomic component of the DR is mediated by the medullary circuitry and preserved in decerebrated animals (12, 24). The afferents of the ethmoidal nerve project to the medullary dorsal horn neurons through the trigeminal ganglion. Medullary dorsal horn neurons issue multiple projections (25). Transsynaptical viral tracing of the ethmoidal nerve projections revealed fibers reaching nucleus tractus solitarii, rostral ventrolateral medulla (RVLM), lateral tegmental field, Kolliker Fuse nucleus, and superior salivatory nucleus (SSN) (1, 26). Sympathetic activation responsible for the pronounced peripheral vasoconstriction seems to be mediated by RVLM (27), which, among others, harbors neurons innervating preganglionic neurons of intermediolateral column of the spinal cord and is critical for the control of sympathetic tone (28). Importantly, stimulation of RVLM produces metabolically independent increase in cerebral blood flow (CBF) (29, 30) and exerts some neuroprotection (Yamamoto and Golanov, unpublished observation). Projections of the medullary dorsal horn neurons receiving ethmoidal nerve afferents are observed in pre-Botzinger and trapezoid nuclei (31), which are known to participate in respiratory regulation (32–34) and may be involved in the apneic component of DR (1). DR-associated bradycardia most probably involves activation of parasympathetic preganglionic neurons of nucleus ambiguus, which receive projections from medullary dorsal horn neurons targeted by ethmoidal afferents (35).

Integral effect of DR is overall decreased oxygen consumption and preservation of vital functioning of heart and brain during apneic period (17, 36–38). DR, an evolutionary ancient mechanism of survival of low-oxygen/anoxic conditions (39–41), presents in all animals (1) and exerts powerful protection against anoxic conditions (42). There are reports of humans who remained submerged under water for prolonged periods of time (over 1 h in some cases) and fully recovered afterward (43–46). Survival after near drowning does not depend on body temperature (45, 47, 48), and the DR is suggested to be an important component of survival (49).

While stimulation of the ethmoidal nerve produces DR (23), its transection does not eliminate DR in rats, suggesting that other branches of the ophthalmic nerve are capable of triggering DR (50). In humans, dipping face into cold water is sufficient to initiate typical DR consisting of hypertension and bradycardia (2, 8, 10, 51, 52). In fact, it was suggested that stimulation of face cold receptors is vital for the “survival” DR response (53). These findings allowed us to hypothesize that forehead stimulation can be neuroprotective. In agreement with this hypothesis, we established that cold or electrical forehead stimulation exerts neuroprotective effects, decreasing the infarction volume induced by permanent middle cerebral artery occlusion in rats (54). We suggested that forehead stimulation is capable to trigger DR and accompanying activation of endogenous neuroprotective system (55). Non-invasive, simply applicable method of activation of the DR opens potential of using its protective properties in clinical settings.

The basic “tri-partite” components of the DR – hypertension, bradycardia, and apnea – seem to be mediated at the medullary level (1, 3, 27). However, this basic circuitry mediating autonomic component of the DR is also under control of suprabulbar structures (56). The suprabulbar components of the DR are not well investigated. Subthalamic vasodilator area (SVA) plays an important role in the hypoxia-induced cerebral vasodilation and affords neuroprotection upon stimulation, as we established earlier (57, 58). We hypothesized that SVA may be involved in suprabulbar regulatory mechanisms of DR. Here, we explored whether cold stimulation of the forehead triggers autonomic responses comparable to those induced by nasal mucosa stimulation with ammonia vapors as a “classic” model of DR and compared the responses to changes evoked by cold stimulation of the glabrous skin of tail base in anesthetized rats, and possible role of SVA in DR mechanisms.

## Materials and Methods

All experiments were performed in accord with NIH “Guide for the care and use of laboratory animals” and approved by the IACUC of the University of Mississippi Medical Center.

### General Procedures

The methods were described in detail in our previous publications (58). In short, experiments were performed in adult male Sprague-Dawley rats (250–300 g), maintained in thermally controlled facilities with 12/12 h light cycle and *ad libitum* access to lab chow and water. Anesthesia was initiated in the induction chamber using 5% isoflurane and maintained during surgery at 2–2.5%. All experiments were conducted under isoflurane level of 1.2–1.5% in mixture of 80% N_2_ and 20% O_2_. Both femoral arteries were cannulated to monitor arterial pressure and to sample blood for blood gasses. Animals were intubated and ventilated using mechanical ventilator at 50–60 strokes/min. Blood gasses were maintained at normal level (pH 7.46 ± 0.023; PaO_2_: 94.3 ± 1.1 mmHg; PaCO_2_: 34.2 ± 0.8 mmHg) (59). Body temperature was maintained at 37°C using feedback-controlled thermoblanket. Following instrumentation, animals were placed in the stereotaxic frame. The calvarium was exposed through the midline cut and the bone was thinned over the area of 3 mm × 4 mm over the parietal cortex to place laser Doppler needle probe (Periflux PF3, Perymed). A stainless steel screw was inserted through the bone extradurally 0.5 mm rostral and 1 mm lateral to bregma for EEG recording. Arterial pressure was recorded using strain-gauge pressure transducer. EEG was recorded monopolarly with the reference electrode placed in the muscle caudally to the midline cut. EEG signal was filtered at 0.1–100 Hz. Laser Doppler probe (0.45 mm diameter) was positioned over the thinned bone over the parietal cortex area avoiding visible large vessels, and drop of paraffin oil was placed under the probe to provide optical contact. The probe was left in place till the end of experiment. Regional CBF was recorded with time constant of 0.2 s and expressed in arbitrary units. After placing the probe, cerebrovascular reactivity was assessed by adding CO_2_ to breathing mixture for ~2 min, which increased PaCO_2_ to ~50–60 mmHg. This maneuver triggered fast elevation of CBF by 60–90%. The test was repeated several times during the experiment. If reactivity was lost, animal was euthanized and excluded from the analysis.

### Cold Stimulation

Animal forehead was shaved and small thermocouple was introduced under the skin at the rostral angle of the cut on the top of the head. To induce cold stimulation, 1,1-difluoroethane (“Canned Air”) was directly applied to the forehead skin for 5 s. 1,1-difluoroethane is a volatile liquid with a boiling point of −25°C. Upon application, it immediately evaporated and decreased under-skin temperature to a minimum of ~12°C by 15 s after the beginning of application. Temperature gradually returned to the baseline of 35.6°C in ~2 min. Identical stimulation has been applied to the base of the tail. There are no known irritation effects of skin application of the 1,1-difluoroethane besides possible “frostbites,” when excessive amount is applied for extended period of time. We did not observe any residual skin effects, such as redness or edema, even after multiple short applications of 1,1-difluoroethane in our experiments.

### Ammonium Application

To introduce ammonia vapors into the nasal cavity polyethylene catheter (PE-50) was introduce into the nasal cavity through the nares until it reached nasopharynx. Piece of cotton saturated with 50% solution of ammonia hydroxide was placed near the nostrils, and gentle suction was applied for 5 s to the external end of the nasal catheter to create a slightly negative pressure in the nasopharynx, allowing ammonia vapors to be sucked into the nasal cavity through the nares (3). Cotton ball was removed while suction was continued to evacuate ammonia hydroxide vapors from the nasal cavity. All tests were applied three times in each animal with the 10 min intervals.

### Excitotoxic Lesion of Subthalamic Vasodilatory Area

The intrinsic neurons of the SVA were bilaterally destroyed with neurotoxin, ibotenic acid. Rats were anesthetized using face mask and placed in stereotaxic frame. Calvarium was exposed, and, through a burr hole, glass micropipette with the tip diameter of 40–50 μm was inserted at 4.8 mm posterior and 1.5 lateral to bregma to the depth of 7.2 mm. Single injection of 3 nmol of IBO in 20 nl of phosphate buffered saline (PBS) was delivered. After injection pipette was kept in place for additional 5 min to avoid backflow. Symmetrical injection on the other side was performed. After wound closure and recovery from anesthesia, animals were kept in the home cage for 5 days before the experiment. Control animals received injection of PBS. As microinjections of PBS into SVA did not affect baseline or cooling and ammonia response the data obtained in this animals were pooled together with naïve animals.

### Histological Procedures

After euthanasia with carbon dioxide, brains were removed and frozen in isopentane and stored at −80°C until analysis. For histological analysis, brains were sectioned at 20 μm thickness at −20°C and stained with thionin. Lesioned sites were identified using anatomical brain atlas (60) (Figure 6).

### Data Collection and Processing

All data were digitized using ADInstruments digitizer and stored for further analysis. Data processing, including fast Fourier transformation (FFT) of EEG, was performed using LabChart software package. Mean arterial pressure (MAP) was calculated according to the formula: 2/3 diastolic pressure + 1/3 systolic pressure. Cerebrovascular resistance (CVR) was calculated as a ratio between MAP and CBF and expressed as percentage of change relative to the baseline. EEG was normalized as percentage of the total power in 0.1–15 Hz interval. For the study purposes, EEG rhythms were defined as follows: delta rhythm – 0.1–3.0 Hz; theta rhythm – 3.1–7.0 Hz; alpha rhythm 7.1–11.0 Hz; beta rhythm 11.1–15 Hz. Data were expressed as mean ± SEM. For statistical analysis, *t*-test for independent and repeated measures and two-way repeated measures ANOVA with Bonferroni *post hoc* multiple comparisons were used (SPSS). Differences were considered significant at *p* < 0.05.

## Results

**Figure 1:**
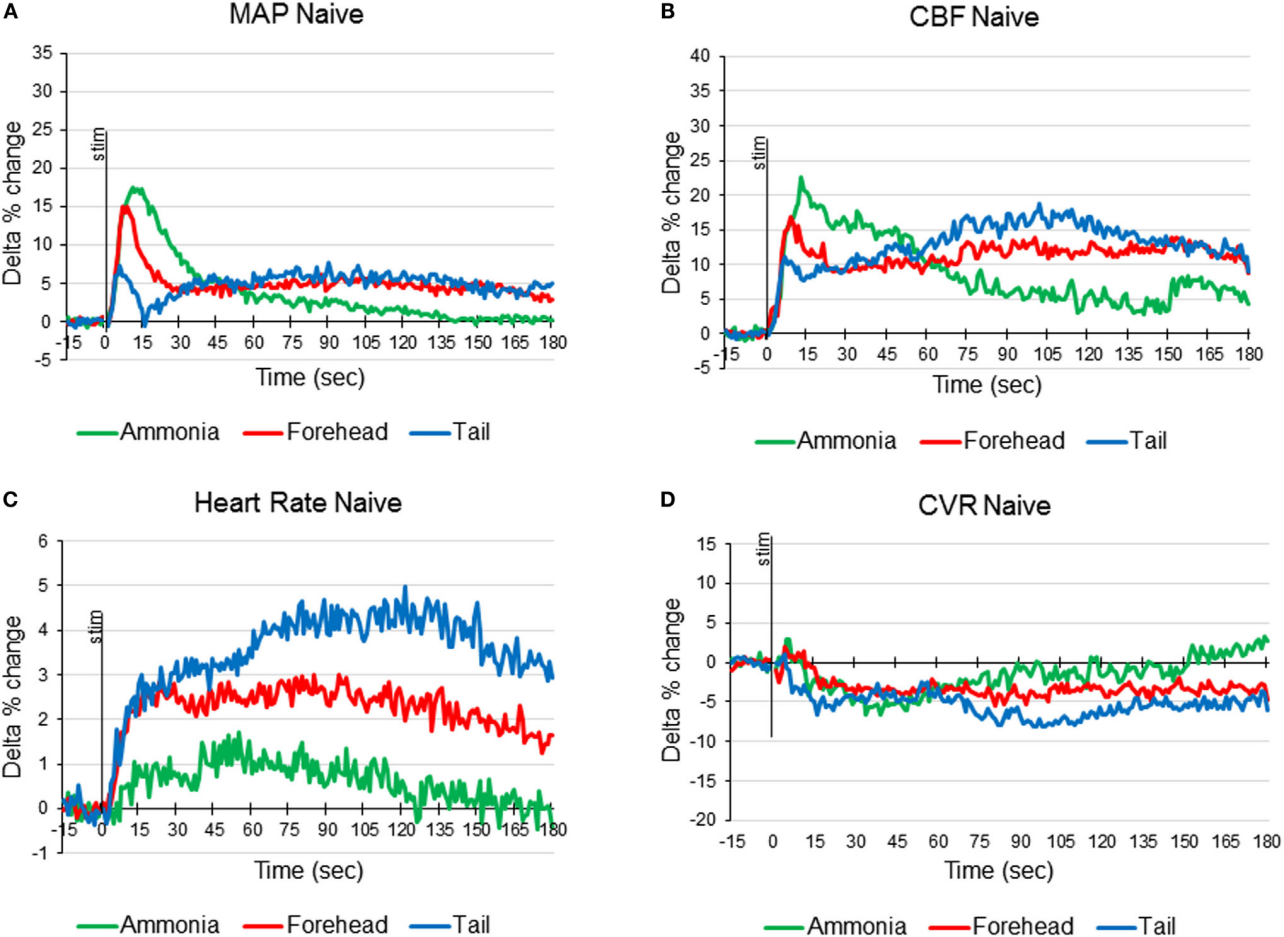
**Averaged responses of mean arterial pressure [MAP (A)], cerebral blood flow [CBF (B)], heart rate (C), and cerebrovascular resistance [CVR (D)] to ammonia vapors instillation into the nose (green line), cold stimulation of the forehead (red line), and cold stimulation of the tail base (blue line) in naïve anesthetized artificially ventilated rats expressed as delta percent change compared with baseline (*n* = 12 animals, 36 tests of each modality)**.

In 12 animals, response to ammonia vapors passage through the nasal cavity has been analyzed. In response to ammonia vapor passage through the nasal cavity, MAP increased by 17.5 ± 3.3% (from 95.6 to 112.5 mmHg, *p* < 0.05) in 11 ± 1 s and returned to the baseline in 146 s (Figures 1A and 4A). The increase in MAP was accompanied by small and slow increase in heart rate (HR), reaching maximum of 1.7 ± 0.4% (from 359.6 to 365.3 beats/min, *p* < 0.05) in 55 ± 4 s (Figures 1C and 4C). In parallel, CBF demonstrated robust increase by 22.8 ± 3.5% reached in 11 ± 1 s (*p* < 0.5, Figures 1 and 4B). Increase in CBF was accompanied by non-significant decrease in CVR by −6.6 ± 3.3% at 34 ± 5 s returning to the baseline in 80 s (Figures 1D and 4D). In response to intranasal ammonia vapor administration, power of EEG delta rhythm significantly decreased by 10.6 ± 5.1% (*p* < 0.05) compared with the background activity. At the same time, theta rhythm power increased by 7.2 ± 3.3% (*p* < 0.05), while alpha and beta rhythm did not change significantly (Figure 3).

**Figure 2:**
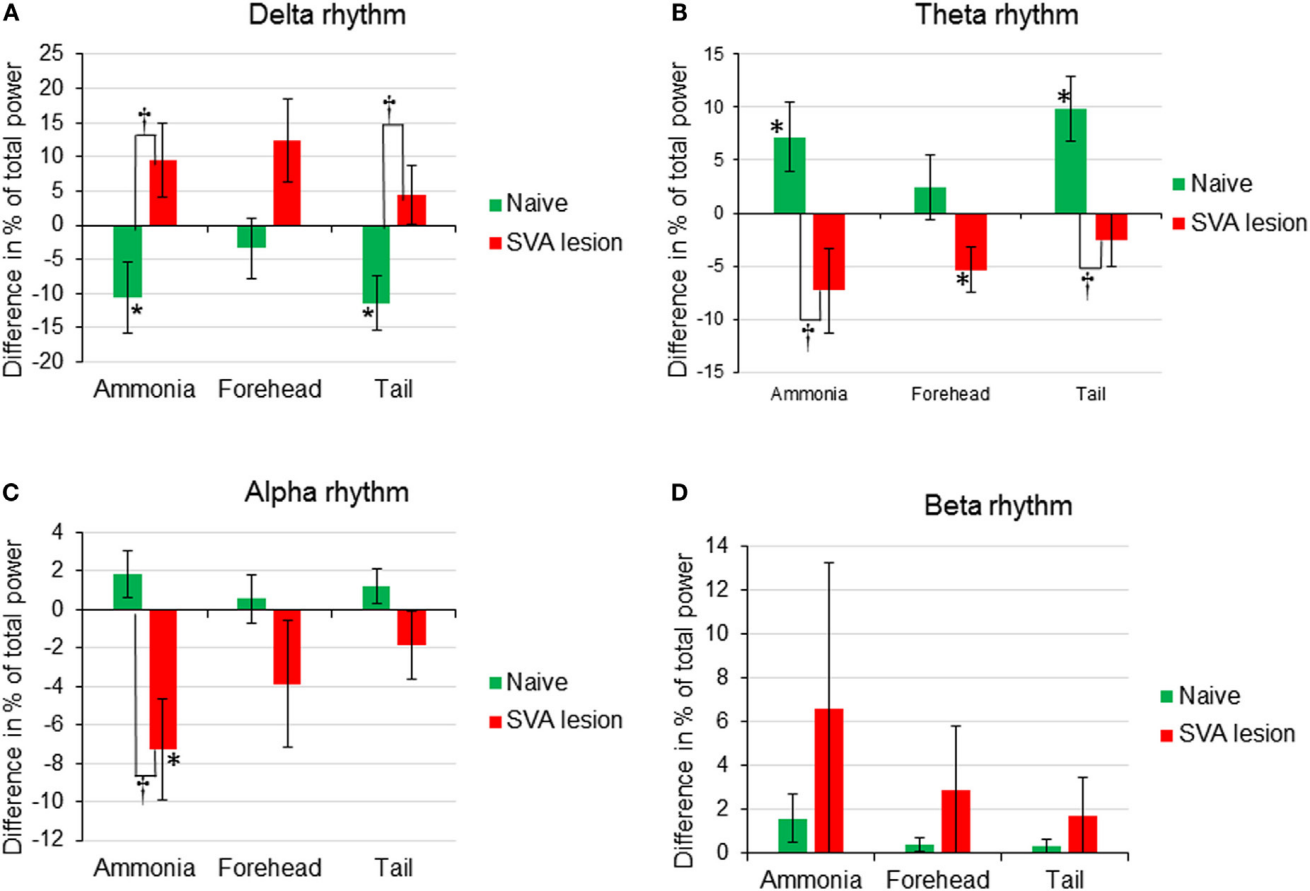
Comparison of changes (difference between percent of total power before and after stimulus) of EEG rhythms [(A) Delta rhythm, (B) Theta rhythm, (C) Alpha rhythm, (D) Beta rhythm] expressed as change in percent of total power in response to nasal ammonia vapor instillation and forehead and tail cold stimulation in naïve (*n* = 12, green bars) and animals after the lesioning of the subthalamic vasodilator area (*n* = 7, red bars), *p < 0.05 significance of the amplitude of the response compared with the baseline, ^†^*p* < 0.05, significance between response in naive and SVA-lesioned animals, error bars – SEM.

**Figure 3:**
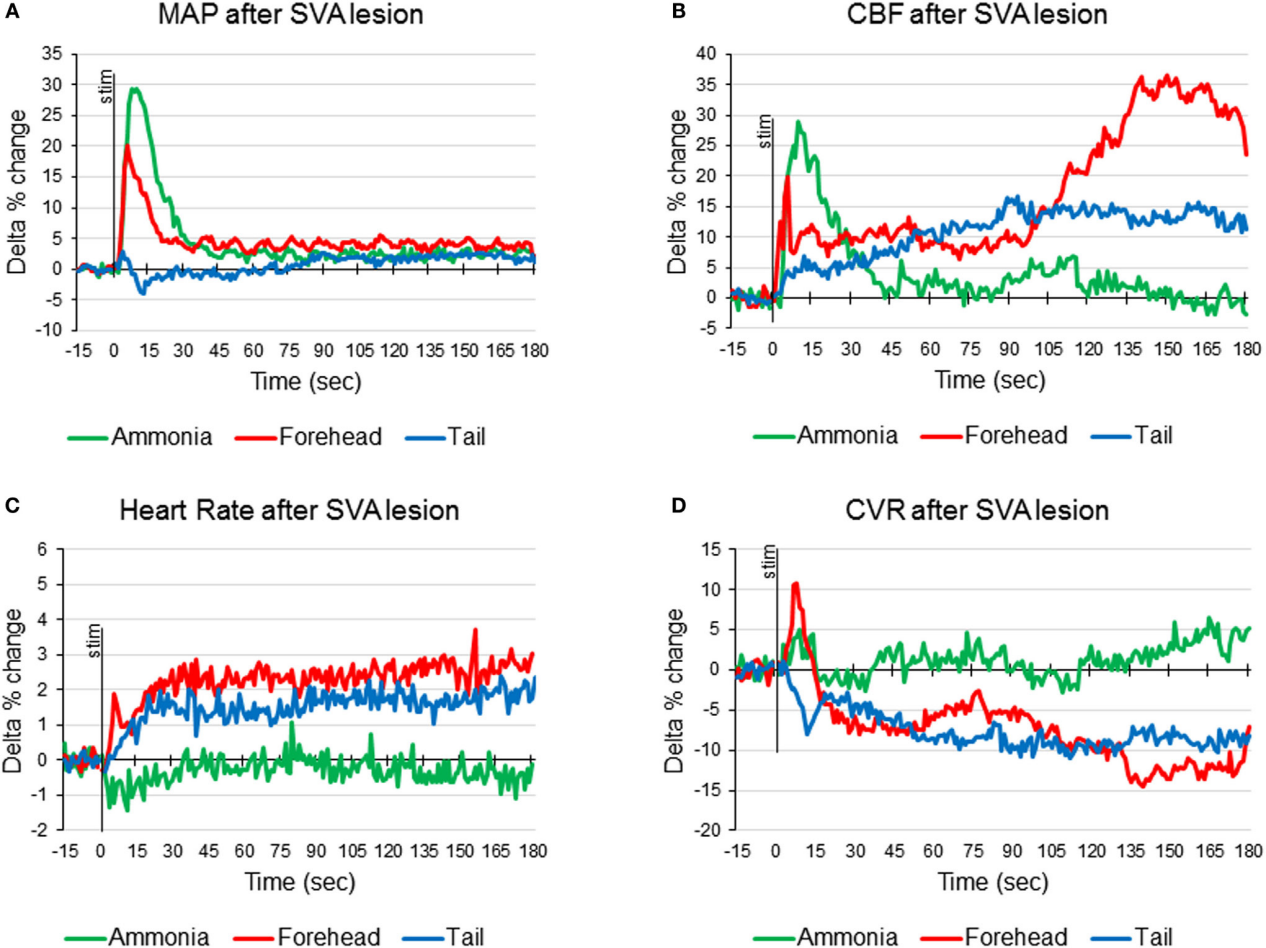
**Averaged responses of mean arterial pressure [MAP (A)], cerebral blood flow [CBF (B)], heart rate (C), and cerebrovascular resistance [CVR (D)], to ammonia vapors instillation into the nose (green line), cold stimulation of the forehead (red line), and cold stimulation of the tail base (blue line) in anesthetized artificially ventilated rats after the lesioning of the subthalamic vasodilator area (SVA) (n = 7 animals, 21 tests of each modality)**.

**Figure 4:**
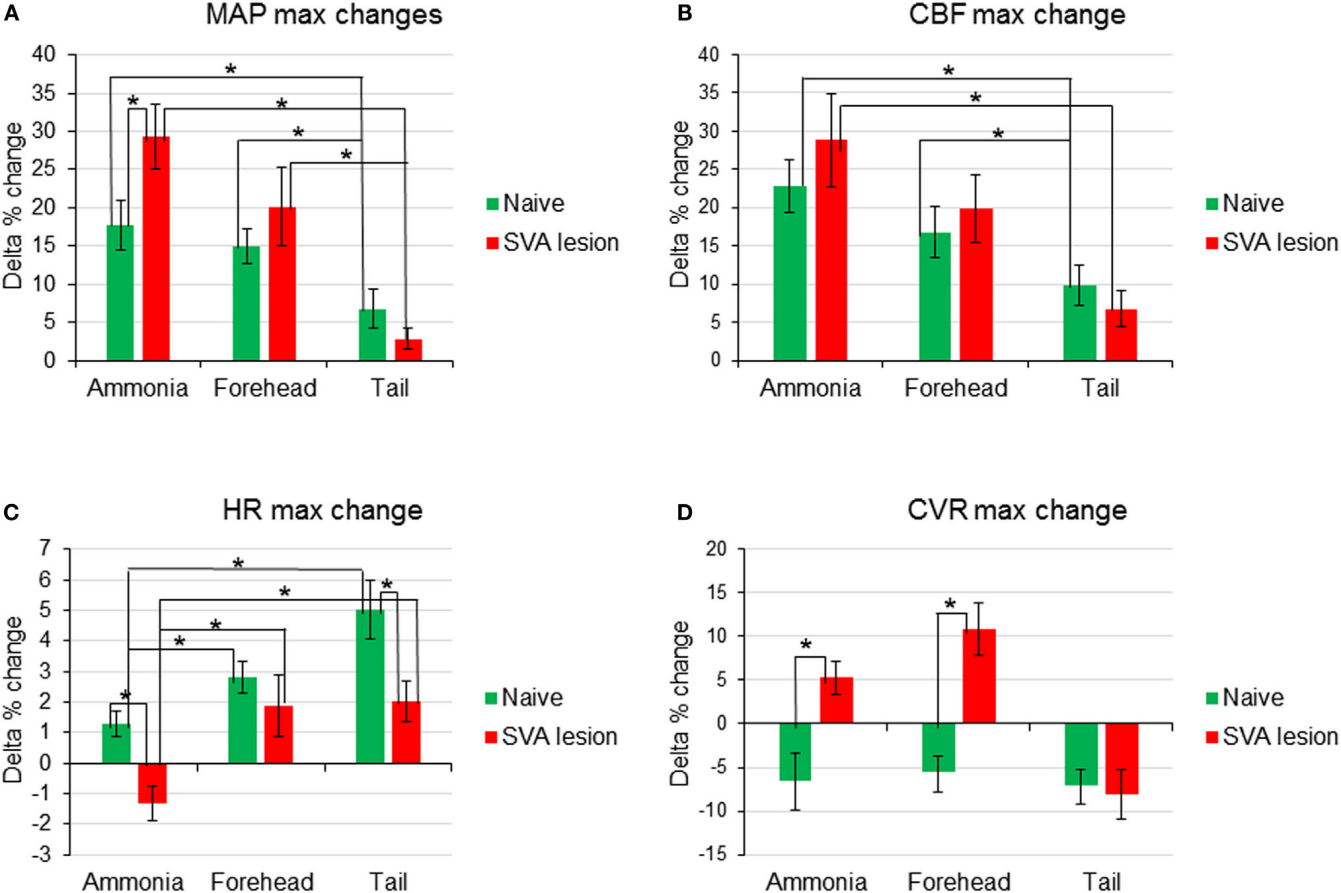
Comparison of changes of the maximum amplitudes of early responses of mean arterial pressure [MAP (A)], cerebral blood flow [CBF (B)], heart rate [HR (C)], and cerebrovascular resistance [CVR (D)] in response to nasal ammonia vapor instillation and forehead and tail cold stimulation in naïve animals (*n* = 12, green bars) and animals after the lesioning of the subthalamic vasodilator area (*n* = 7, red bars), *p < 0.05, error bars – SEM.

In the same animals, response to the forehead cooling was tested. Five-second application of cold stimulus to the forehead triggered decrease of the subcutaneous temperature from 35°C to a minimum of 12°C reached in 19 s after the initiation of cooling and returned to the baseline by 157 s. In response to decreased forehead temperature MAP raised by 15.0 ± 2.1% (*p* < 0.05, from 92.8 to 106.7 mmHg) in 7 s when subcutaneous temperature decreased to 16.7°C (Figures 1A and 4A). Increase in MAP was comparable to that observed in response to passage of ammonia (*p* > 0.05). In parallel, HR increased by 2.8 ± 0.5% (*p* < 0.05, from 353.4 to 363.2 beats/min) reaching maximum at 23 s (Figures 1C and 4C). CBF started to increase within 2 s of the beginning of cooling and reached maximum of 16.8 ± 3.4% (*p* < 0.05) in 9 s. After insignificant increase at 10 s, CVR decreased significantly by 5.5 ± 2.2% (*p* < 0.05) at 81 s (Figures 1D and 4D). All parameters returned to the baseline within 5 min. Forehead cooling did not affect EEG significantly (Figure 3).

Comparable 5-s cooling of the tail base triggered biphasic increase in MAP. First, fast increase reached 7.2 ± 2.5% in 6 s (from 92.3 ± 2.4 to 98.9 ± 4.1 mmHg, *p* < 0.05;) followed by the secondary delayed increase by 7.7 ± 2.6% (to 101.7 ± 3.5 mmHg, *p* < 0.05) at 90 s (Figures 1 and 4A). MAP returned to the baseline in 5 min. Increases in MAP response to tail base cooling was significantly smaller than responses to ammonia passage or forehead cooling evoked responses. HR in response to tail base cooling was gradual, and, after fast initial increase, it continued to rise reaching maximum of 4.2 ± 1.8% (from 354.4 ± 5.7 to 370.0 ± 9.6 beats/min, *p* < 0.05) by 120 s and returned to the baseline also in 5 min (Figures 1 and 4C). CBF increase likewise was biphasic: after peaking in 7 s by 9.8 ± 2.7% (*p* < 0.05), it continued to rise after the short decrease and reached 18.1 ± 3.1% (*p* < 0.05) by 102 s and returned to the baseline in parallel with MAP (Figures 1 and 4B). CVR also demonstrate biphasic changes similar to MAP and CBF, the first decrease of −7.0 ± 2.2% (non-significant, n.s.) in 16 s after the stimulus onset was followed by the secondary slightly deeper decrease by −8.1 ± 3.1% (*p* < 0.05) at 111 s. CVR returned to the baseline in parallel with CBF in 5 min (Figures 1 and 4D). In response to tail base stimulation, delta rhythm was significantly depressed by 11.1 ± 3.2% (*p* < 0.05) and theta rhythm increased by 9.9 ± 4.4% (*p* < 0.05). Alpha and beta rhythm were not affected significantly (Figure 3).

### Effects of SVA Lesions

**Figure 5:**
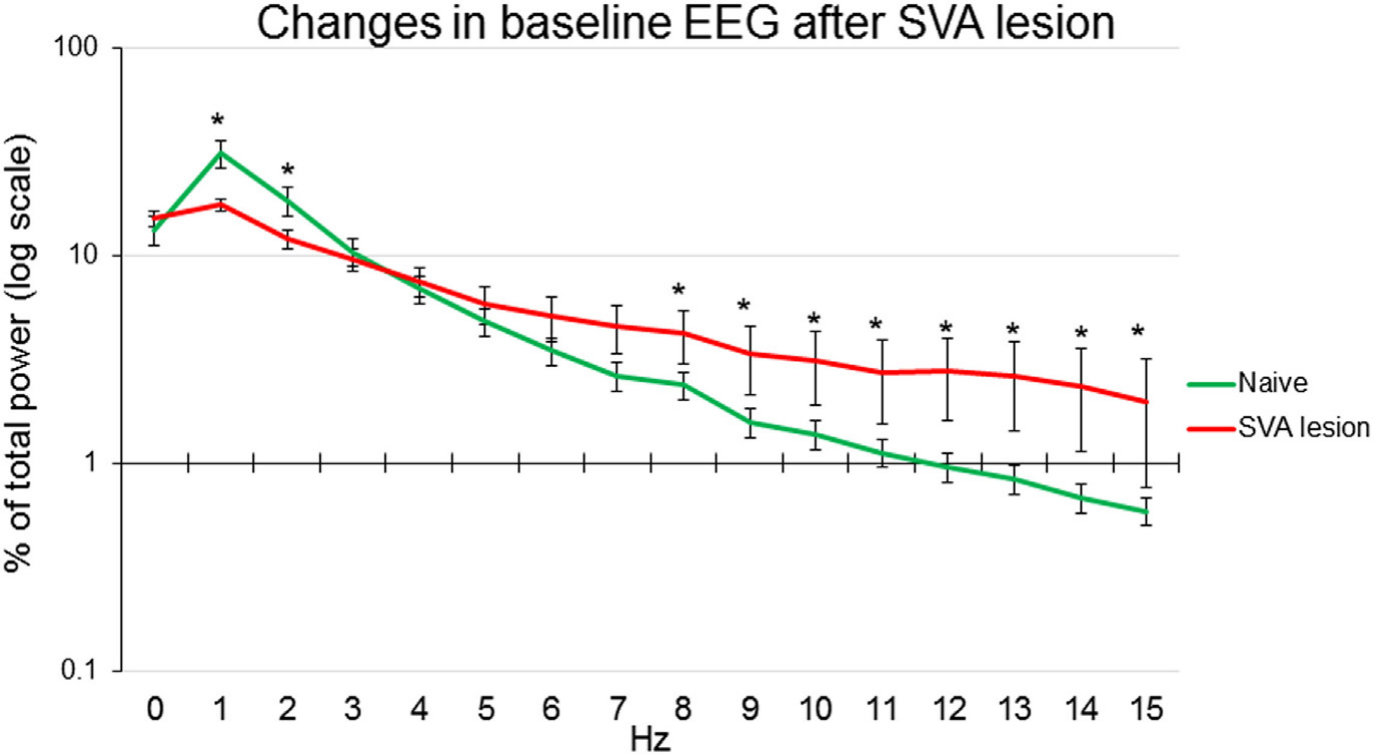
Power of EEG components expressed as percent of total power in naïve anesthetized artificially ventilated animals (*n* = 12, green line) and in anesthetized artificially ventilated animals after lesion of subthalamic vasodilator area (*n* = 7, red line), *p < 0.05 comparison between naïve and lesioned animals, error bars – SEM.

**Figure 6:**
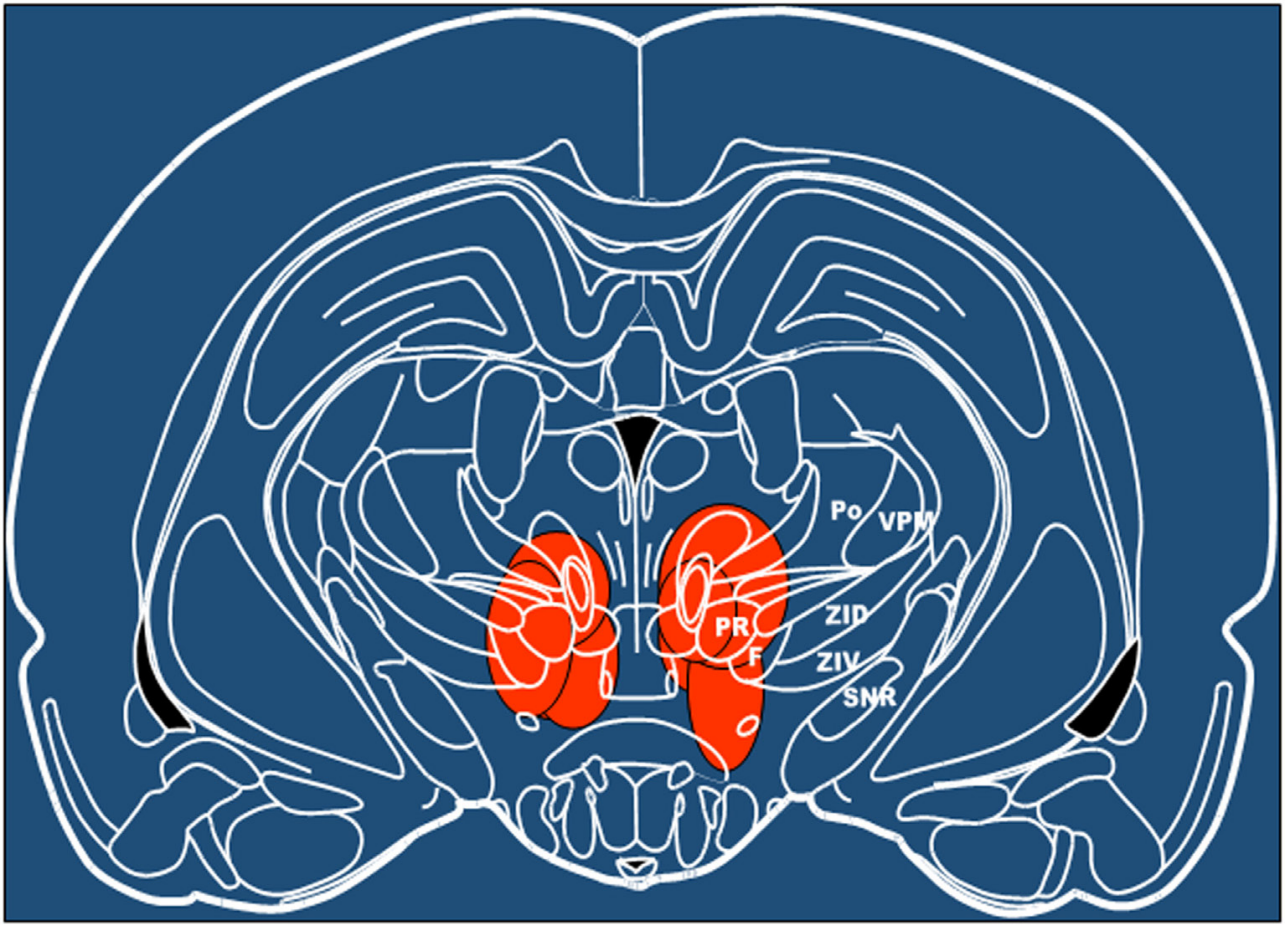
**Schematic presentation of areas lesioned by ibotenic acid injection based on the histological analysis**. Level −4.8 mm from bregma (60). F, nucleus of the fields of Forel; Po, posterior thalamic nuclear group; PR, prerubral field; SNR, substantia nigra, reticular part; VPM, ventral posteromedial thalamic nucleus; ZID, zona incerta, dorsal part; ZIV, zona incerta, ventral part.

In seven other animals, SVA was lesioned by intraparenchymal injection of ibotenic acid. Histological identification of the localization of lesion of subthalamic vasodilatory area established gliosis in SVA and in the immediate vicinity, including mediate pole of zona incerta, prerubral nucleus, and field of Forel (Figure 6). Only animals that demonstrated gliosis in the SVA area were included in the analysis. Baseline absolute values of MAP were significantly (*p* < 0.05) higher: 107.3 ± 4.0 mmHg in SVA-lesioned animals compared with 92.7 ± 3.1 mmHg in naïve animals. HR did not differ significantly between naïve and SVA-lesioned animals: 354.5 ± 8.1 beats/min and 360.1 ± 9.1 beats/min, respectively. Baseline EEG was significantly affected by SVA lesion. Background delta rhythm power decreased by 18.2 ± 2.3%, alpha and beta rhythm powers increased by 9.0 ± 2.4% and 6.7 ± 1.9%, respectively (*p* < 0.05), while theta rhythm power did not change significantly (Figure 5).

In SVA-lesioned animals, MAP change in response to ammonia passage through the nasal cavities was significantly (*p* < 0.05) facilitated compared with non-lesioned animals and reached 29.3 ± 4.2% (from 106.8 ± 3.9 to 138.5 ± 6.2 mmHg) with that comparable to non-lesioned animals latency of 10 s (Figures 3A and 4A). MAP returned to the baseline within 5 min. HR decreased within 4 s by 1.3 ± 0.6% (from 365.3 to 361.6 beats/min). While the decrease was not significant compared with the baseline it differed significantly when compared with naïve animals, which demonstrated increase in HR (Figures 3C and 4C). CBF response was facilitated reaching 28.6 ± 6.1% (*p* < 0.05), which was not significantly different from the response in naïve animals. However, CBF returned to the baseline significantly faster than in naïve animals within 41 s (Figures 3B and 4B). Following SVA lesion, CVR demonstrated non-significant short increase of 5.3 ± 1.2% in 9 s (Figures 3D and 4D). In response to ammonia passage, only alpha rhythm was significantly suppressed by 7.3 ± 1.2% (*p* < 0.05), while power of other rhythms did not reach level of significance (Figure 2).

In response to the forehead cooling in SVA-lesioned animals, MAP increased comparable to the response observed in naïve animals, but, like response to ammonia, was slightly higher and reached 20.2 ± 5.1% (*p* < 0.05) (from 105.7 ± 3.6 to 126.9 ± 4.2 mmHg) of baseline in 6 s and returned to the baseline in 24 s (Figures 3A and 4A). HR in response to the forehead stimulation increased in 6 s by 1.9 ± 0.8% (n.s.) (from 355.61 ± 13.1 to 361.7 ± 10.8 beats/min) followed by secondary increase by 2.5 ± 1.0% (n.s.) (to 363.8 ± 9.5 beats/min) at 40 s which also did not significantly differ from other stimuli and slowly returned to the baseline in 5 min (Figures 3C and 4C). CBF, in response to stimulation, increased in 6 s in parallel to MAP, reaching 19.9 ± 4.5% with secondary increase up to 36.2 ± 6.5% at 136 s and slowly returned to the baseline in 5 min (Figures 3B and 4B). CVR changes in SVA-lesioned animals were amplified compared with naïve animals. CVR in parallel with CBF increased acutely to 10.8 ± 3.4% (*p* < 0.05) by 8 s and decreased by −7.9 ± 2.6 (*p* < 0.05) at 32 s and further dropped to a minimum of −14.5 ± 4.5% at 140 s, returning to the baseline in 5 min (Figures 3D and 4D). Forehead cooling induced suppression of theta rhythm by 6.4 ± 1.8% (*p* < 0.05), while other rhythms remained unchanged (Figure 2).

Response to tail base cold stimulation also was affected in SVA-lesioned animals. Increase in MAP was attenuated and changes were non-significant with the slight increase of 2.3 ± 0.8% (from 107.6 ± 1.2 to 110.1 ± 5.3 mmHg) within 4 s, followed by drop and return to the baseline in 3 min (Figures 3A and 4A). HR increased by 1.9 ± 1.0% at 24 s (from 359.9 ± 9.3 to 365.1 ± 9.6 beats/min), while not reaching significance compared with the background. However, it was significantly (*p* < 0.05) less than in naïve animals (Figures 3C and 4C). CBF response also was attenuated. Initial peak of increase of 6.8 ± 1.4% was reached in 12 s with the secondary increase at 93 s to 16.8 ± % (*p* < 0.05), with gradual return to the baseline in 5 min (Figures 1, 2 and 4B). In parallel, CVR dropped to −8.1 ± % with the further decrease to −14.0 ± 3.5 (*p* < 0.05) in 112 s and returned to the baseline in 5 min (Figure, 1, 2, 4D). Cold stimulation of the tail base failed to significantly modify EEG in SVA-lesioned animals (Figure 2).

## Discussion

### The Model

In our experiments, we explored whether cold stimulation of the forehead is capable to induce DR. We compared autonomic responses triggered by stimulation of the nasal mucosa with ammonia and by cold stimulation of the forehead or the glabrous skin of the tail base. Changes in AP and CBF evoked by ammonia vapors instillation into the nasal cavity or by cold stimulation of the forehead were similar. Both responses, however, differed significantly from those induced by cold stimulation of glabrous skin of the tail base.

It is generally accepted that stimulation of the nasal mucosa triggers DR (1, 61–63). The prototypic DR observed in marine animals can be reproduced in other animals, including birds and terrestrial animals. It consists of characteristic and unique triad: hypertension, bradycardia, and apnea, which result from the simultaneous coordinated activation of sympathetic and parasympathetic systems (1, 11, 12). Our experiments demonstrated that MAP, HR, CBF, and CVR responses to the application of the stimuli of different modality, but within the zone innervated by the trigeminal nerve, were comparable. At the same time, the stimuli of the same modality (cold) applied to areas innervated by different nerves, forehead and tale base, produced different responses. These observations suggest that activation of trigeminal system evokes autonomic responses, which differ from responses triggered from other areas. Along with that, some common features between tail and forehead cooling induced responses suggest the existence of shared mechanisms probably related to excitation of somatic cold receptors. Because nasal mucosa stimulation-induced response is considered archetypal DR, we suggest that forehead stimulation also triggers response, which is close if not identical to DR. This conjecture finds support in the observations that, in humans, cold face stimulation triggers autonomic changes, hypertension, and bradycardia, similar to the DR (2, 8, 10, 51, 52).

It is thought that cold stimulation of the ophthalmic branch of trigeminal nerve initiates DR (2). Cold stimulation of the forehead and ammonia nasal mucosal stimulation in our experiments triggered autonomic responses typical for the DR. However, we did not observe bradycardic component of the DR. The weak tachycardic response observed by us probably occurred due to artificial ventilation and isoflurane anesthesia. Lung inflation attenuates bradycardia during DR (64), and isoflurane is capable of decreasing the parasympathetic cardiac drive (65). Simultaneous use of isoflurane in combination with mechanical ventilation in our experiments may negate bradycardic response. This speculation is supported by our observations of pronounced bradycardic responses accompanied by hypertension to electrical stimulation of the forehead in spontaneously respiring rats under isoflurane anesthesia (66, 67). These findings also indicated that apnea plays an important role in the bradycardic component of the DR (68). Overall, it is possible to conclude that forehead cold stimulation triggers autonomic response comparable to DR.

Studies of the mechanisms of the DR are complicated by the various problems related to difficulties of working with diving animals or use of voluntary or forced diving in terrestrial animals (9). Our model allows studying mechanisms of DR in laboratory conditions using various physiological approaches. Use of artificial ventilation provides advantages to explore the mechanism of the DR. First, it obviates heart–lung reflexes (69), which, while a part of the “normal” DR, complicate studies of central mechanisms responsible for the initiation of the DR. Second, it allows to maintain normal partial pressure of blood gasses and avoids superimposition of chemoreflexes, which also complicate studies of the central mechanism of the DR. Our model of the DR using forehead cold stimulation offers advantages to dissect its central mechanisms.

### Changes in Cerebral Blood Flow

It is generally assumed that blood flow to the brain increases during the DR (11, 12). However, limited amount of data are available on the changes of CBF during the DR. Blood flow velocity in the middle cerebral artery in humans increases in response to face cold test (70, 71). Direct stimulation of nasociliary nerve triggers transient increase in CBF (72). Other studies failed to demonstrated changes in CBF related to trigeminal system activation (73, 74). In our model, ammonia nasal stimulation and cold forehead stimulation triggered robust increase in CBF, suggesting that trigeminal stimulation triggers increase in CBF independent of modality. The central mechanisms of the DR include activation of RVLM and nucleus tractus solitarius (NTS) (1). These structures are capable not only to regulate the activity of sympathetic and parasympathetic systems but also induce global neurogenic increase in CBF (75–77). Neurogenic origin of CBF increase is evidenced by the decrease in CVR because mechanisms of autoregulation maintain stable CBF in face of increased MAP by increasing CVR (78). It is conceivable that activation of RVLM also initiates increase in CBF as a part of the DR. However, limited decrease in CVR suggests that, in our model, neurogenic cerebrovasodilation is not the major component of CBF increase and results also from the increase in MAP mediated by excitation of locus coeruleus and Kolliker Fuse nuclei (18). This suggestion is further confirmed by the fact that lesion of SVA mediating RVLM-induced CBF increase (58) failed to do so in our experiments.

### Role of Subthalamic Vasodilatory Area

Autonomic components of the DR are mediated by the medullary circuitry (7, 12, 24) as the afferents of the trigeminal ophthalmic branch through the trigeminal ganglion project to NTS, RVLM, lateral tegmental field, Kolliker Fuse nucleus, and SSN (26, 31). Peripheral autonomic reflexes comprising DR (nasotrigeminal reflex) in traditional sense seem to be mediated by the medulla and spinal cord (3). However, this basic circuitry mediating autonomic component of the DR is also under control of suprabulbar structures (56) and regulates brain activity, brain vasculature, and CBF. SVA activation triggers neurogenic metabolically independent increase in CBF evidenced by the decrease in CVR without affecting AP. It also participates in cerebrovasodilation induced by hypoxia (58). Moreover, stimulation of the SVA affords neuroprotection (57). We hypothesized that SVA may participate in the DR mechanisms. However, lesion of SVA did not reverse but augmented increase in MAP and CBF in response to stimulation of nasal mucosa or forehead. HR increase was suppressed (or unchanged in case of the forehead stimulation) and, in response to ammonia application, was even reversed becoming slightly bradycardic. Tail base responses, on the opposite, were suppressed. Interestingly, MAP increase evoked by tail base stimulation was also suppressed. Amplified decrease in delayed, secondary CVR in response to tail stimulation suggests increased neurogenic cerebrovasodilation in response to tail base stimulation following SVA lesion. To summarize, it is possible to suggest that SVA may attenuate sympathetic activation of the heart rate and somatically (tail) induced MAP. At the same time, trigeminally induced changes in AP seem to be potentiated, which explains amplified CBF response. Secondary delayed increase in CBF following forehead cold stimulation and after SVA lesion may relate to release of vasodilatory mediators, such as NO, or prostaglandins and requires further investigation. To conclude, it seems that SVA does not participate directly in trigeminal or somatosensory cerebrovasodilation, but rather modulates these responses. It is conceivable that MAP increase-related increase in CBF is sufficient to provide additional blood supply without neurogenic cerebrovasodilation, which is consistent with the DR-associated “centralization” of circulation. Whether SVA participates in DR-induced neuroprotection (66, 67) remains to be established.

### EEG Activity

Limited data are available on the EEG changes accompanying DR. In seals, EEG was changing from alpha low voltage activity to prevalence of high voltage slow waves (79). Apnea alone does not produce significant changes in EEG (80). Trigeminal stimulation has been proposed to suppress seizure-like EEG synchronization (81, 82). In our experiments, ammonia nasal stimulation and tail base stimulation shifted power of EEG frequencies from delta to theta rhythms. Delta rhythm is generally associated with deep non-REM sleep and reflects decreased brain metabolic activity (83). Theta rhythm is observed in various conditions, including general anesthesia, attention, and activity (84). There seems to be two different types of theta rhythm. The first type has higher frequency, can be blocked with atropine, and relates to repeated voluntary behavior. The second type – atropine insensitive – is of lower frequency and relates to general anesthesia or behavioral immobility (85). It was demonstrated that orexin A, synthesized by neurons of lateral hypothalamus promotes wakefulness. Intracerebroventricular administration of orexin A leads to decrease in the power of delta rhythm and simultaneous increase in the power of theta rhythm (86). In our experiments, we observed shift from delta to theta rhythm in response to ammonia nasal stimulation and tail base stimulation. This observation suggests that these stimuli exert short arousal-like effect. Because of anesthesia, full arousal did not occur as evidenced by the lack of alpha desynchronization. Intralaminar and midline thalamic nuclei participate in arousal processes (87). SVA is localized to the posterior midline subthalamic area. Stimulation of this functionally defined area triggers appearance of synchronized EEG activity (58). In our experiments, lesion of SVA significantly suppressed background delta and theta rhythm, while facilitating expression of alpha and beta rhythms. These observations suggest that SVA participates in the maintenance of the specific level of synchronization. At the same time, SVA lesion reversed EEG shift in response to stimuli: power of slow delta rhythm increased while power of theta rhythm decreased in response to stimuli. These data suggest that SVA participates in the regulation of the synchronization–desynchronization balance. Because lesion of SVA amplified synchronization responses, it seems that SVA is not the leading source of synchronization but rather is a modulator of other structures, which induce cortical synchronization.

## Conclusion

Our experiments demonstrated similarities between ammonia-induced and forehead cooling-induced response pattern of MAP and CBF. At the same time, these responses differed from somatically (tail base) cold-induced response. These observations suggest that activation of the ophthalmic branch of the trigeminal nerve triggers specific physiological changes compatible with the pattern of “classic” DR observed in animals and humans. Experiments with the lesion of SVA demonstrated that, while SVA does not mediate trigeminal of somatically induced cerebrovasodilation, it modulates these responses and participates in EEG changes accompanying DR. Further investigations of the role of SVA and other elements of endogenous neuroprotective system in DR related neuroprotection are granted.

Diving response directed toward survival of the anoxic periods “is the most powerful and enigmatic reflex” (1). Activation of “oxygen conserving” DR may have beneficial effects in various conditions, such as obstructive sleep apnea, stroke, TBI, and hemorrhagic shock. Our recent experiments indicate that forehead stimulation, indeed, affords neuroprotection following ischemic stroke (54) and traumatic brain injury (66, 67). The present study demonstrates that forehead stimulation triggers response comparable to DR. Further understanding of this complex phenomenon will allow the development of new therapeutic approaches for the various pathologies.

## Author Contributions

EG designed and performed experiments, analyzed data, and prepared the manuscript. JS performed experiments. GB prepared and edited the manuscript.

## Conflict of Interest Statement

The authors declare that the research was conducted in the absence of any commercial or financial relationships that could be construed as a potential conflict of interest.
